# A pilot and feasibility study of a randomized clinical trial testing a self-compassion intervention aimed to increase physical activity behaviour among people with prediabetes

**DOI:** 10.1186/s40814-022-01072-6

**Published:** 2022-05-27

**Authors:** Alana K. Signore, Mary E. Jung, Brittany Semenchuk, Sasha M. Kullman, Olivia Tefft, Sandra Webber, Leah J. Ferguson, Kent Kowalski, Michelle Fortier, Jon McGavock, Rashid Ahmed, Marion Orr, Shaelyn Strachan

**Affiliations:** 1 Faculty of Kinesiology and Recreation Management, University of Manitoba, Winnipeg, MB R3T 2N2 Canada; 2 School of Health and Exercise Sciences, University of British Columbia, Kelowna, BC V1V 1V7 Canada; 3 Applied Health Sciences, University of Manitoba, Winnipeg, MB R3E 0T6 Canada; 4 College of Rehabilitation Sciences, University of Manitoba, Winnipeg, MB R3E 0T6 Canada; 5 College of Kinesiology, University of Saskatchewan, Saskatoon, SK S7N 5B2 Canada; 6 School of Human Kinetics, University of Ottawa, Ottawa, ON K1N 6N5 Canada; 7 Faculty of Health Sciences, University of Manitoba, Winnipeg, MB R3E 3P5 Canada; 8 College of Nursing and Professional Disciplines, University of North Dakota, Grand Forks, ND 58202 USA; 9 Inner Compass Counselling, Winnipeg, MB R3G 2X6 Canada

**Keywords:** Mindfulness, Self-kindness, Common humanity, Self-regulation, Behaviour change, Acceptability

## Abstract

**Background:**

Seventy-five per cent of individuals with prediabetes will eventually be diagnosed with type 2 diabetes. Physical activity is a cornerstone in reducing type 2 diabetes risk but can be a challenging behaviour to adopt for those living with prediabetes. Individuals with prediabetes experience difficult emotions associated with being at risk for a chronic disease, which can undermine self-regulation. Self-compassion enhances self-regulation because it mitigates difficult emotions and promotes adaptive coping. We performed a pilot randomized controlled trial to determine the feasibility and acceptability of a self-compassion informed intervention to increase physical activity for persons with prediabetes.

**Methods:**

This explanatory mixed methods study tested the feasibility and acceptability of a two-arm, randomized, single-blind, actively controlled, 6-week online intervention. Using a 1:1 allocation ratio, participants (identified as people with prediabetes, low physical activity, and low self-compassion) were randomized to a self-compassion (*M*_age_ = 60.22 years) or control condition (*M*_age_ = 56.13 years). All participants received behaviour change education (e.g. SMART goals, action-coping planning) and either other health knowledge (control condition: e.g. sleep, benefits of water) or self-compassion training (intervention condition: practising mindfulness, writing a letter to themselves offering the same support that they would offer to a friend). The primary outcome was to determine the feasibility and acceptability of the trial. To be considered feasible, our outcomes needed to meet or surpass our pre-determined criteria (e.g. *time for group formation*: 14–20 participants per month). Feasibility was assessed by examining the recruitment rates, retention, adherence, fidelity, and capacity. Semi-structured interviews were conducted with participants to determine trial acceptability. As a secondary purpose, we examined the means on key study variables (secondary and exploratory variables; see Table 1) at all planned time points (baseline, intervention-end, 6- and 12-week follow-up) to identify if they are suitable to include in the efficacy trial (see Additional Table 3).

**Results:**

Eighteen participants were screened and randomized to one of two conditions. Retention, instructor fidelity, safety, capacity, adherence to most of the study aspects, and acceptability by participants and facilitators all met the criteria for feasibility. Recruitment rate, process time, and adherence to home practice were below our criteria, and we offer ways to address these shortcomings for the efficacy trial.

**Conclusion:**

The results from this study suggest that it should be feasible to deliver our intervention while highlighting the alterations to components that may be altered when delivering the efficacy trial. We outline our changes which should improve and enhance the feasibility and acceptability of our planned intervention. Funding for this study was from the Canadian Institutes of Health Research (CIHR).

**Trial registration:**

ClinicalTrials.gov, NCT04402710. Registered on 09 April 2020.

**Supplementary Information:**

The online version contains supplementary material available at 10.1186/s40814-022-01072-6.

## Key messages regarding feasibility

- What uncertainties existed regarding the feasibility?
  How many participants would be interested/eligible to participate? What would be the reasons for participant dropout? How much home practice would participants engage in? How adherent will participants be towards study components? What would the intervention’s acceptability look like in terms of content and logistics?
- What are the key feasibility findings?
  Most outcomes met our pre-determined criteria which indicate that the planned future trial should be feasible and acceptable with minor changes; recruitment rate, process time, and adherence to home practice fell below our pre-determined criteria, and we propose suggestions to improve these shortcomings for the larger efficacy trial. The means of key variables suggest that all measures will be appropriate for the larger trial. Overall, the findings from the present study offer support for the planned efficacy trial to be successful.
- What are the implications of the feasibility findings for the design of the main study?
  Alterations to some components aim to ensure that the procedures of a larger trial should be feasible and acceptable. Strategies will be implemented to improve the intervention components of recruitment rate, process time, home practice, and increasing time for recruitment and completion of eligibility steps. For instance, in the future efficacy trial, we will highlight the significance of home practice to participants while increasing accountability through class discussions of home practice. Additional alterations will be made to the larger trial based on participant feedback/acceptability (e.g. more/longer sessions).

## Introduction

Prediabetes, a chronic condition defined as having blood glucose levels that are elevated to just below the threshold for a type 2 diabetes (T2D) diagnosis [1], affects 352 million people worldwide [2]. Prediabetes is associated with an increased risk for nephropathy [3], cardiovascular disease, all-cause mortality [4], and mental health issues (i.e. anxiety, stress [5]). In addition, approximately 75% of people with prediabetes develop T2D [6]. Intensive lifestyle modification is efficacious for preventing progression to T2D among people with prediabetes. For example, in a large-scale randomized controlled trial (RCT), an intensive lifestyle intervention that targeted 150 min of physical activity per week and healthy eating led to 7% weight loss and reduced progression to T2D by 58% over 3 years, compared to the control arm [7]. Other researchers (e.g. [8]) found that daily physical activity substantially reduces the risk of T2D, independent of diet and weight loss. Despite this evidence, the majority of individuals with prediabetes do not engage in the recommended 150 min per week of moderate to vigorous physical activity [9]. Further, the majority of adults in one study who received a chronic disease diagnosis did not adopt and/or maintain health behaviours, including physical activity, over 14 years post-diagnosis [10]. Inactivity among people with prediabetes may have negative effects on their health status if changes are not made. Although previous physical activity interventions have shown improvements in physical activity among individuals with prediabetes (e.g. [11]), a key piece that is missing from previous interventions is how to effectively manage one’s emotional response to prediabetes diagnoses and their struggles to becoming more active.

Engaging in physical activity appears to be challenging for people with prediabetes and this may be in part due to the complex nature of the behaviour which requires many self-regulation skills to maintain (e.g. realistic goal-setting, monitoring goal progress [12]). Moreover, individuals with prediabetes face the risk of chronic diseases [13], which can lead to difficult emotions (e.g. fear, shame) that impact their ability to engage in physical activity [14]. Indeed, these emotional states can lead to impaired decision-making [15] and less goal-directed behaviour [16]. It is important to find strategies that help individuals with prediabetes self-regulate their physical activity and cope with the difficult emotions they may experience after their diagnosis in order to increase and maintain their physical activity.

Self-compassion, defined as the orientation to care for oneself during challenging times [17], may help individuals with prediabetes self-regulate their physical activity and cope with difficult emotions. Through the three components of self-compassion, an individual recognizes they are not alone in their imperfections (i.e. common humanity), learns to forgive and be kind to themselves despite imperfections (i.e. self-kindness), and pays attention to their experiences without ruminating or disengaging from them (i.e. mindfulness). Theoretically, self-compassion has both a comforting “yin” side and an action-oriented “yang” side [18]. In times of suffering, the yin of self-compassion allows an individual to provide themselves with comfort and validation [19]. Meanwhile, using the yang of self-compassion individuals can “act” in the world by protecting, providing, and motivating themselves to do what is in their best interest [19]. Therefore, through activating the yin of self-compassion, individuals should be able to cope with difficult emotions experienced after a prediabetes diagnosis; when activating the yang of self-compassion, they should take action such as prioritizing physical activity.

Indeed, research supports both the role of the yin and yang of self-compassion in coping with emotions and the self-regulation of health behaviours. In support of the yin of self-compassion, studies have shown that self-compassion is positively associated with adaptive emotional regulation (e.g. [19]) and negatively related to difficult emotions (e.g. guilt, shame) in response to exercise lapses [20]. Among eight independent samples, Sirois and colleagues [21] found a positive relationship between self-compassion and health behaviours, including physical activity, which was mediated by its inverse relationship with negative affect. In support of the yang of self-compassion, this construct has also been positively associated with setting goals focused on health and well-being [22], a proactive health focus [23], and self-improvement motivation [24]. Further, self-compassion has been associated with additional aspects of self-regulation including goal re-engagement and situational intrinsic motivation [20]. In a systematic review and meta-analysis (*N* = 5622), Wong and colleagues [25] found that self-compassion was positively associated with physical activity behaviour regulation (*r* = .29, *p* < .01) and daily physical activity (*r* = .26, *p* < 0.01).

Self-compassion has been found to be helpful for people with chronic conditions. Semenchuk and colleagues [23] shared with middle-aged women their moderate- to high-risk status for cardiovascular disease. Among these women, self-compassion was associated with adaptive emotional and behavioural responses to the news such as less rumination and an increased likelihood of seeking out information about their condition [23]. Further, a scoping review of eleven studies (*N* = 3488) found that self-compassion is associated with and also leads to adaptive behavioural and affective responses among individuals with diabetes (i.e. prediabetes, type 1, T2D, and gestational diabetes) [26]. For instance, self-compassion was negatively associated with unpleasant affective states (e.g. diabetes distress [25]). The self-compassion interventions included in the review found significant reductions in HbA1c levels (i.e. blood glucose), diabetes distress, and depression, compared to controls (e.g. [27]), which were maintained at 3 months post-intervention. A limitation noted within this review was that there was only a single study [14] focused on people with *prediabetes*. This study qualitatively explored how people diagnosed with prediabetes reacted emotionally to their diagnosis [14]. Morgan and colleagues [26] call for more *intervention* research examining the role of self-compassion in facilitating health behaviours and adaptive emotional responses among individuals with prediabetes; increasing health behaviour among this population is important because individuals can positively impact their risk for T2D and other health complications [2].

Though self-compassion interventions have proven effective at conveying a positive change in a variety of outcomes (e.g. depression [27]), including health behaviours (e.g. smoking cessation [28]), only one intervention to our knowledge has examined the effect of self-compassion on physical activity [29]. This intervention focused on whether self-compassion and mindfulness training led to increases on a scale that assessed *self-reported* health behaviours (i.e. nutrition, stress management, and physical activity) among twenty-four community adults. The intervention led to significant increases in self-compassion and some health behaviours including self-reported leisure time physical activity. No study has examined whether a self-compassion intervention leads to increases in objectively measured physical activity in a larger sample. Further, these were healthy community adults—no research has examined how self-compassion training can help individuals who are at risk of chronic diseases to increase their physical activity. Our study seeks to address these gaps in the literature. As self-compassion is associated with coping with health risk information [23], self-regulatory challenges [20], and engagement in health behaviours [29], individuals with prediabetes who are taught how to treat themselves compassionately should be in a better position to make changes to their physical activity. Furthermore, self-compassion may augment traditional self-regulation training that can lead to increases in physical activity (e.g. [30]) by teaching people with prediabetes how to support themselves as they cope with their disease risk and try to increase their physical activity.

## Study purpose

The purpose of this mixed-method pilot RCT was to determine if a self-compassion intervention designed to increase physical activity among people with prediabetes is feasible and acceptable. The information gleaned from this pilot study will inform a future trial where we will test whether supplementing 6 weeks of online behaviour change education (e.g. goal-setting [30]) with self-compassion training leads to larger increases in physical activity than behaviour change education alone among people with prediabetes. Pilot studies that focus on feasibility and acceptability are an important step prior to efficacy trials to improve the quality of future trials. Pilot studies of this type make it less likely that unforeseen circumstances will de-rail an entire intervention effort [31]. We hypothesized that the present pilot intervention would be feasible and acceptable according to the predetermined criteria.

## Methods

### Study design

This was a randomized, explanatory mixed-methods pilot and feasibility study. Assessments occurred at eligibility, baseline, intervention-end, and 6- and 12-week follow-up. This trial was registered with ClinicalTrials.gov (NCT04402710), and the Consolidated Standards Reporting Trials guidelines informed our reporting (CONSORT [32, 33]). This study received ethics approval at Canadian University. Following sample size recommendations for pilot feasibility studies [34], we aimed to recruit 20 participants (10 in each arm).

### Participants and recruitment

Participants were recruited through media advertisements (e.g. Facebook, radio, news). Interested participants completed an online questionnaire as a first eligibility screening step where they had to satisfy the following criteria: had moderate-to-high risk scores for T2D determined by the CANRISK assessment tool [35], low self-report physical activity (i.e. ≤ 600 MET minutes a week according to the International Physical Activity Questionnaire (IPAQ [35])), between 40 and 74 years old, not receiving medical treatment or behaviour change education for T2D, could safely engage in physical activity (PAR-Q+ [36]), available for all testing and sessions, and below the average population score on self-compassion [37]. Participants who satisfied these criteria were asked to provide online study consent and wear an accelerometer for 8 days to confirm their self-reported physical activity; participants were deemed eligible if they accumulated less than 150 min of sporadic moderate-to-vigorous physical activity per week (i.e. Canadian Physical Activity Guidelines [38]). Eligible and consenting participants were emailed the baseline questionnaire which consisted of demographic and covariate measures.

### Randomization, group formation, and blinding

Participants were allocated to the intervention or control arms via a randomization sequence using a 1:1 allocation ratio conducted by a statistician removed from the study operations. Because both groups received education, participants in the control condition were unaware that they were in the control group. E-mails containing assessments (i.e. questionnaires) were sent to participants by the coordinator, whereas the study personnel (i.e. research assistants, principal investigator) who interacted with participants (e.g. accelerometer drop-offs, online one-on-one sessions) were blinded to participant allocation.

### The intervention

The online intervention involved a 60-min online one-on-one meeting with a research assistant (i.e. session 1) followed by five group education sessions (sessions 2–6) each 1 week apart. During the first one-on-one meeting, the research assistant shared the participant’s T2D risk score (i.e. moderate or high risk of T2D) and discussed the behaviours that could lower T2D risk [35], including meeting the physical activity guidelines (i.e. 150 min of moderate to vigorous physical activity per week [38]), describing how physical activity could decrease T2D risk, and discussing their past experience with physical activity. All individual meetings followed the same script for both conditions and represented what a patient diagnosed with prediabetes would ideally experience with a diabetes educator (i.e. ideal care [34]). The subsequent five online group sessions covered behaviour change topics followed by either self-compassion (intervention group) or health topics unrelated to the study outcomes (control group). Participants in both groups were encouraged to complete weekly “home practice” activities facilitated by a workbook which were related to weekly topics. Furthermore, participants were reminded about the different components of the study (e.g. home practice, upcoming sessions) and were asked to report how much home practice they completed through a text messaging system. A website was created for each group (and secure to only each group) where participants could find additional information about session content, places to exercise, and free exercise videos. Two facilitators (i.e. PhD student and post-doctoral fellow) conducted the five group sessions; both had expertise in Mindful Self-Compassion training [39] and experience in conducting behaviour change interventions. Additional resources provided to participants can be found in the additional file (see Additional Table 1). Given that the intervention was offered online, participants completed and signed a confidentiality agreement to ensure that all personal information/identification (e.g. health information, name, age) shared in the group sessions was kept private and confidential. To further ensure participant privacy, participants were reminded to partake in the session in a private room and were given an alternative option to communicate with the facilitator by using the private chat.

#### Behaviour change training (sessions 2–6)

All participants received 45 min of theory-based physical activity behaviour change education, based on effective physical activity interventions among people with prediabetes (e.g. [40]). Behaviour change topics included goal-setting, self-monitoring, self-efficacy, physical activity barriers, enjoying physical activity, and relapse prevention.

#### Self-compassion content (sessions 2–6)

For the second half of sessions 2–6, participants in the self-compassion condition received 45 min of self-compassion training. Adapted from the Mindful Self-Compassion training created by Neff and Germer [39], participants learned how to apply self-compassion to their prediabetes and physical activity experience through strategies shown to increase self-compassion [39]. Some examples of activities included having participants apply the three aspects of self-compassion to one’s diabetes risk and having them identify their self-critical voice and replace it with their self-compassionate voice. Topics included an introduction to self-compassion, the yin and yang of self-compassion, mindfulness, meeting difficult emotions, and embracing the good in life.

#### Control content (sessions 2–6)

For the second half of sessions 2–6, participants in the control condition received information on health topics unrelated to physical activity. Control material was similar in activity nature and duration to the self-compassion condition in order to match the amount and type of attention provided in the self-compassion condition; this allowed us to rule out possible effects from activities or attention [41]. Topics included sleep, screen time, antibiotic use, benefits of water, and benefits of vitamin D.

### Feasibility

We were guided by Thabane and colleagues’ [42] framework to identify our feasibility outcomes. Quantitative assessments were used to determine feasibility. Feasibility outcomes included recruitment rate, process time, retention rates, adherence rates, instructor fidelity, and capacity. The a priori criteria were set for all outcomes in order to determine whether changes needed to be made for our future efficacy trial [42]. Qualitative methods were used to explore intervention acceptability and intervention safety.

#### Recruitment

Our key outcomes were *time for group formation (i.e.* our criteria were set as 14–20 participants per month) and *process time*; the time it takes to enrol a participant into a group which our criterion was set at 2–3 weeks (i.e. completion of eligibility questionnaire, PARQ+, 8 days for accelerometer wear). For planning purposes, we observed the (i) number of interested people, (ii) percentage of people screened for eligibility, (iii) number of eligible participants recruited to the trial, (v) reasons for ineligibility, and (vi) the success of recruitment strategies.

#### Retention rate

*Retention rate* was defined as the number of participants who completed the trial relative to those who dropped out. The criteria for drop-out rate at intervention-end were 15–20% and an additional 10% drop-out at 6- and 12-week follow-up [28, 40].

#### Adherence rates

*Adherence rates* were determined through class attendance, responses to text messages, and accelerometer wear. The criteria were defined as (i) 80% home practice completion, (ii) 80% intervention attendance, (iii) accelerometer wear time of 4 days, 10 h per day or greater [43], and (iv) 80% of participants wearing an accelerometer.

#### Instructor fidelity

*Instructor fidelity* was assessed against our criterion of facilitators (i) presenting 95% of all intervention topics and (ii) achieving a total assessment score (by the study principal investigator) of four out of five on adherence to the planned intervention, presentation skills (e.g. pacing, tone), and communication skills.

#### Capacity

*Capacity*, or how many study personnel and time was required for study tasks, was judged against our criterion of personnel hours falling within our estimated budget.

### Acceptability

To establish the *acceptability* and psychological *safety* of the intervention, participants and facilitators were asked open-ended questions during an online exit interview or open-ended questionnaire. For instance, to assess psychological safety, participants were asked, “Please explain whether there was anything in the group sessions that made you feel comfortable, welcomed, or connected to the group”. Our criteria for psychological safety were set at 100%. Interview guides were produced which followed “the four-phase process to interview protocol refinement” framework by Castillo-Montoya [44]. The interview guide can be found in the Additional file Table 2.

### Secondary and exploratory outcome measures

For feasibility purposes, we evaluated the procedure participants would endure in the larger efficacy trial, including the completion of scales (i.e. receptivity, comprehension, and tolerability [42]). Physical activity was determined from waist-mounted accelerometer worn for a period of 8 days. The full list of secondary and exploratory outcomes is described below in Table 1.

**Table 1:** Secondary and exploratory outcomes

Measure	Purpose	Subscales	Scoring and reliability	Cronbach’s alpha
Self-Compassion Scale (26 items [45])	Measured at all time points to determine self-compassion levels.	Self-kindness vs. self-judgement, common humanity vs. isolation, and mindfulness vs. over-identification	A total score of self-compassion was determined by adding the means of all six sub-scales together and dividing by six [45]. Scores from this scale have good test-retest reliability (*r* = .80–.93) with high internal consistency (*α* = .92 [45]). A sample item includes “When things are going badly for me, I see the difficulties as part of life that everyone goes through”.	Baseline (*α* = .86), post (*α* = .95), 6 weeks (*α* = .93), 12 weeks (*α* = .92)
Daily Minutes of MVPA^a^ (ActiGraph GT3X+ accelerometer [46])	Measured at all time points to determine an objective assessment of daily minutes of MVPA was measured using a hip-worn ActiGraph GT3X+ accelerometer [46] during waking hours for 8 days; also used to determine eligibility.		Freedson cut-points were used [47], and 8 days of accelerometer wear time was chosen as it allowed for a 1-day “wash out” period to account for accelerometer reactivity (i.e. the first day was not used [48]). The ActiGraph GT3X+ specifically measured the participants’ volume and intensity of physical activity [49] and accelerations in a standing, lying, or sitting position [46].	n/a
Short-form IPAQ^b^ (4 items [50])	Measured at all time points to determine participants’ self-report physical activity behaviours (i.e. walking, moderate- and vigorous-intensity activities over the last 7 days); also used to determine eligibility.		Participants reported the number of days they engaged in each intensity and the average duration of each session. This scale has shown evidence of validity with moderate to high reliability (0.71–0.89 [51]). The total scores were created for each intensity separately (i.e. multiplying each intensity by its respective MET value; a total score was then calculated by summing all METs from each intensity. A sample item includes “During the last 7 days, how many days of 10 min or more did you complete of vigorous physical activities like heavy lifting, digging, aerobics, or fast bicycling?”	n/a
Negative Affect Scale (20 items each [52])	Measured at all time points to determine participants’ emotions relative to their (i) T2D^c^ risk and (ii) physical activity engagement.	Sadness, anger, embarrassment, anxiety, and incompetence	A total score was created for each subscale. Versions of this scale demonstrate acceptable reliability (*α* = .75 [52]). A sample item includes “After hearing about your type 2 diabetes risk/when thinking about my engagement in physical activity, to what degree do you feel sad?”	Baseline (*α* = .82–.95), post (*α* = .92–.96), 6 weeks (*α* = .75–.96), 12 weeks (*α* = .64–.95)
Exercise Barrier Scale (14 items [53])	To determine the extent to which participants relate to barriers to exercise at baseline and intervention-end.		A total score was created; a higher score indicates greater barriers to exercise [53]. Scores from this scale are reliable and valid with Cronbach’s alpha of *α* = .87 [54]. A sample item includes “Exercising takes too much of my time”.	Baseline (*α* = .84), post (*α* = .88)
Cognitive Emotion Regulation Questionnaire (36 items [55])	Measured at all time points to determine the extent to which participants used certain cognitive-emotional regulation strategies.	Self-blame, other-blame, rumination, catastrophizing, putting into perspective, positive refocusing, positive reappraisal, acceptance, and planning	Individual subscale scores were summed; a higher subscale score indicates greater use of that coping strategy [55]. Scores from the total scale exhibited good internal consistency (*α* = 0.92 [55]). A sample item includes “I think that I cannot change anything about it.”	Baseline (*α* = .31–.93), post (*α* = .61–.95), 6 weeks (*α* = .64–.95), 12 weeks (*α* = .63–.95)
Health Promoting Lifestyle Profile II (52 items [56])	Measured at all time points to determine the extent participants engage in health-promoting behaviours.	Spiritual growth, interpersonal relations, nutrition, physical activity, health responsibility, and stress management	The mean score was calculated for each subscale. This scale has demonstrated high internal consistency (*α* = .94) with alpha coefficients for the subscales ranging from .79 to .87 [56]. Participants were asked to respond to the frequency they engage in different behaviours such as “Discuss my problems and concerns with people close to me”.	Baseline (*α* = .75–.94), post (*α* = .68–.87), 6 weeks (*α* = .78–.89), 12 weeks (*α* = .73–.94)
Additional items	Three additional items created for the present study were included to identify what helped participants increase their physical activity, cope with their prediabetes, and whether they were receiving other information beyond the current intervention. These items were measured at intervention-end.		Items: (i) “What part of the intervention helped you the most when trying to increase your physical activity?”, (ii) “What part of the intervention helped you the most when trying to cope with your prediabetes diagnosis?” and (iii) “At any point throughout this intervention, did you enrol in any education programmes, other than this one, to help you become more physically active or to address your prediabetes?” If they respond yes, they will be asked to please specify.	n/a

^a^*MVPA* Moderate to vigorous physical activity

^b^International Physical Activity Questionnaire

^c^Type 2 diabetes

### Timing of outcome measures

Participants were asked to wear an accelerometer for 8 days and complete an online questionnaire about the key study variables at baseline, immediately after the intervention, and at 6- and 12-week post-intervention. Online interviews were conducted on Zoom (i.e. video conferencing software) post-intervention with interested participants and facilitators to gather information related to intervention acceptability and psychological safety. Participants were given the opportunity to answer the same open-ended questions through an online survey if they preferred an alternative format. Participants were provided a $10 Amazon gift card for each assessment (i.e. questionnaire) and group session they completed throughout the study. All participants were eligible to obtain a total amount of $100 worth of Amazon gift cards.

### Statistical analysis

Descriptive statistics were used to examine the means and standard deviations (when applicable) of primary outcomes related to the feasibility of the intervention. Acceptability and psychological safety from the online interviews were determined by using a deductive thematic analysis [57]. All interviews were transcribed, and accompanying categories and themes were created [57, 58]. To stay within the confines of a pilot feasibility study [59], we report the means and standard deviations of measures at all time points for both the control and the intervention to examine participant interpretation and ceiling effects to determine their appropriateness for use in the planned efficacy trial [31]. The mean changes were not examined given that this study was underpowered to detect differences between the groups or pre-post comparisons which would be inconclusive and potentially misleading [31, 59].

## Results

### Feasibility

Data were collected between August 7, 2020, and February 10, 2021. Recruitment occurred between August 7 and September 30 mainly through Facebook (51.7%) and television news features (27.6%). Instagram (3.5%), a university publication (6.9%), and word of mouth each resulted in some recruitment (10.4%). We did not meet our a priori criteria of recruiting 14–20 participants in the first month. Rather, it took 7 and 8 weeks to recruit our minimum (fourteen) and maximum (twenty) participants, respectively. The process time took 24.67 days for participants to complete all eligibility components which was over our 2–3-week criterion.

**Fig. 1:**
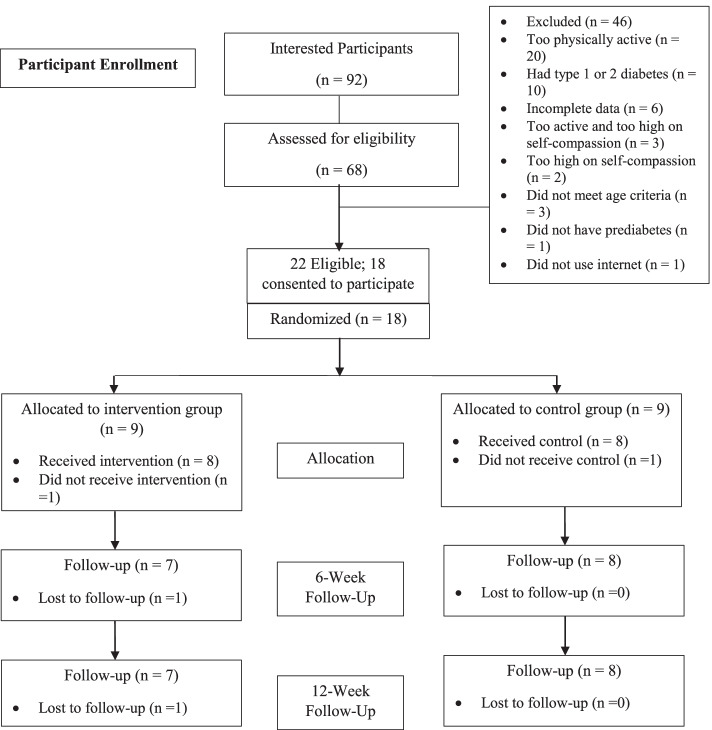
CONSORT flow chart. This is a diagram displaying the participant numbers and flow at each phase of the study from interested participants to a 12-week follow-up

Table 2 shows the baseline demographic and clinical information while Fig. 1 shows the participant study flow. Study interest was expressed by 92 people, 68 of whom engaged in eligibility screening. Overall, 22 people were eligible which translates into 23.9% eligibility among those who expressed interest and 32.4% among those who were screened. Twenty of the 46 ineligible people were too physically active, 10 had diabetes, 6 had incomplete data, 3 were both too active and high on self-compassion, 2 were too high on self-compassion only, 3 did not meet the age criteria, 1 did not have prediabetes, and 1 did not use the Internet. Eighteen of the 22 eligible people (81.8%) agreed to participate, consented, and were randomized to a condition. Online individual meetings (session 1) occurred between September 9 and October 10, 2020; 5 weekly online group intervention sessions ran from October 13 to November 10, 2020. The 6- and 12-week assessments occurred during the weeks of December 16–23, 2020,^1^ and February 3–10, 2021, respectively. Thirteen of the eighteen participants (seven from the control group; six from the intervention group) took part in the virtual exit interview which occurred between November 13 and November 26, 2020. In addition, one participant from the intervention group chose to respond to the open-ended exit questionnaire.

**Table 2:** Participant baseline demographic and clinical information

Variables	Intervention group (*n* = 8)	Control group (*n* = 8)
Mean age in years	60.22	56.13
Sex assigned at birth		
Male	12.5%	12.5%
Female	87.5%	87.5%
How do you describe yourself?		
Male	12.5%	12.5%
Female	87.5%	87.5%
How do you think people would describe your appearance, style, or dress?		
Very feminine	25.0%	25.0%
Mostly feminine	50.0%	50.0%
Somewhat feminine	12.5%	12.5%
Mostly masculine	12.5%	
Very masculine		12.5%
How do you think people would describe your mannerisms?		
Very feminine	12.5%	25.0%
Mostly feminine	62.5%	50.0%
Somewhat feminine	12.5%	12.5%
Mostly masculine	12.5%	
Very masculine		12.5%
Education		
Some high school		25.0%
High school	12.5%	
Some college or university	25.0%	25.0%
A college degree	12.5%	12.5%
An undergraduate university degree	37.5%	37.5%
A doctorate	12.5%	
Ethnicity		
Caucasian	87.5%	100.0%
Aboriginal First Nations	12.5%	
Identify as indigenous person		
Yes	12.5%	
No	87.5%	100.0%
Not a member of a racialized community in Canada	100.0%	100.0%
Relationship status		
Single	37.5%	25.0%
Common-law	12.5%	
Married	12.5%	62.5%
Divorced	25.0%	12.5%
Widowed	12.5%	
Employment status		
Employed full time	37.5%	50.0%
Employed part time	37.5%	12.5%
Self-employed		12.5%
Out of work	12.5%	12.5%
Retired	12.5%	12.5%
CANRISK assessment		
Mean CANRISK score	41.13	43.50
BMI		
Black (BMI 35 and over)	50.0%	37.5%
White (BMI less than 25)	12.5%	
Dark grey (BMI 30 to 34)	37.5%	50.0%
Light grey (BMI 25 to 29)		12.5%
Family history of T2D		
Yes	62.5%	75.0%
No	37.5%	25.0%

Table 3 describes the retention and adherence information. Dropout occurred equally in both groups and occurred for personal (e.g. family issues) and technical reasons. With the exception of completion of home practice, all of our feasibility criteria were met.

**Table 3:** Retention and adherence information

Retention rates	Results	Criteria	Criteria met	Criteria not met
Drop-out at intervention-end	11.11%	15–20%	X	
Drop-out at 6- and 12-week follow-up	8.3%	10%	X	
Reasons for drop-out	Loss of computer/internet; personal reasons	N/A	N/A	N/A
Adherence rates				
Class attendance	98.9%	80%	X	
Home practice completion	7.06/10 (70.6%)—an average of 61.3% was completed by the intervention group; an average of 79.1% was completed by the control group.	80%		X
Accelerometer adherence of 4 days, 10 h	100% at baseline and 12-week follow-up; 84.6% adherence at 6-week follow-up	80%	X	
Participants wearing accelerometer	83.3%	80%	X	

For fidelity purposes, the principal investigator assessed four individual meetings (22%), one for each research assistant, and two group sessions (20%), one session for each facilitator. Fidelity to the intervention exceeded our criterion of four or higher. Facilitators also completed the *facilitator checklists* after each group session; both covered 100% of the prepared content which met our criterion of 90–95%. Finally, the hours required for research personnel to complete all study tasks fell within our capacity criterion.

The means for key efficacy variables for all time points are included in the additional files in Table 3. Baseline means were examined for participant interpretation and ceiling effects to determine their appropriateness for use in the planned efficacy trial (e.g. [30]). Inspection of the physical activity self-report data at follow-up time points revealed an issue with interpretation for some participants. A closer inspection of that measure revealed an error in wording (i.e. we used the word bouts instead of days when asking about physical activity in the IPAQ). No ceiling effects were found.

### Acceptability

Facilitators noted that some session topics raised issues with some participants. For instance, the control facilitator mentioned that the topic of antibiotic resistance led to some strong negative opinions which led to some tension within the group. The topic of “exercise enjoyment” was also not well received by some. For instance, some participants felt that exercise could never be enjoyable. Despite these two concerns expressed by the facilitators, both also noted that the class discussions were valuable. Participants seemed to enjoy discussing their journey to becoming more physically active while also learning a lot from other peoples’ experiences. Data from facilitator exit interviews as well as participant exit surveys and interviews suggest that our planned intervention should be acceptable to individuals with prediabetes. Several themes were identified: positive experiences, receptiveness to study aspects, and new additions to the study. Entangled within each theme were participants’ suggestions for change. Participant quotes related to each theme can be found in Table 4.

**Table 4:** Participant quotes related to themes

Theme	Quotes
Acceptability	“…the antibiotics one, where it almost felt like it was getting a little opinionated…” (control facilitator)
	“it was that topic [exercise enjoyment] I think that some people were like ‘nope, I can’t see how this could ever be something I could enjoy’” (intervention facilitator)
	“…people [were] talking about their goals and their background with exercise and trying to bounce ideas off each other” (intervention facilitator)
Group interaction/common humanity	“so I think one big benefit was the human aspect, right? So, listening to other people saying, ‘I didn’t have a good week’ or ‘I had all these plans for this week, but they kind of fell through’. So, kind of just the reminder that you know you’re human and you don’t have to beat yourself up, you just have to say ‘okay well that didn’t work, I’m going to try harder next week…kind of looking at the human aspect of it all” (participant #3)
	“to know that other people are having the same difficulties makes you not feel like you’re so alone in dealing with it” (participant #11)
	“even just hearing others’ experiences made a big difference” (participant #80)
	“Just the positively, it was very positive experience… so that’s what I like.”
Changed perspective and understanding	“… really, I’m in control of this, and I can do this. I can change these things and I can do this. Whereas prior to that, it was more a matter of, well I was looking more at the obstacles and the challenges instead of looking at, again, if I can do this small change, I can do this small change, I can do this small change; I have these four small things and now I have a big change” (participant #11)
	“it was a reminder and reinforcement of how important it is to exercise. What it does for the mind, the body, the spirit” (participant #16)
	“just recognizing and knowing that even short bouts of exercise can make a difference... that really helped to motivate me to go ‘ok you know what. No more excuses!’” (participant #80).
Changed behaviour	“I finally started going to the gym that I had signed [up] for months ago. And for the first time ever in my life, I’m 63, and for the first time ever in my life, because I’ve joined many gyms and I actually started to enjoy it!” (participant #53)
	“I haven’t been spending as much time sitting in front of the tv during the day. I’m actually making more fresh meals and doing more things and spending more time outside – things like that. So, I might not be moving as fast and making huge leaps, but I feel like those are the steps that we need to get to where we’re going” (participant #69)
	“well I think being more mindful for myself and kinder to myself” (participant #75)
Receptiveness to session content	“I think for me, it was almost the first or second week where we made some goals for ourselves and saw what the barriers were. I think actually sitting down and writing those things down had a huge impact” (participant #69)
	“I think the whole concept of self-compassion, not being so hard on yourself or so judgmental, is a useful one” (participant #50)
	“like the one where you have to soothe yourself and all that. Like I’ve never done that. Ever. So, I found that sort of awkward, but I can see the value in doing that” (participant #66)
	“I enjoyed all of them [control topics]. I have a particular interest in this kind of thing, so I was quite familiar with a lot of the material, but it was really good to refresh and there were aspects of the presentations that I was not aware were specifically helpful for people with prediabetes or even diabetes” (participant #94)
Receptiveness to structure and format	“I think it was a nice number. It wouldn’t have hurt if there were a couple more, but I think it was alright” (participant #53).
	“I would have spread the individual sessions out to 8 or 10 weeks rather than having just 6” (participant #94).
	“I just prefer in person because I think you bond even better with the group when it’s in person. But I could see that some people would prefer doing it over the computer” (participant #66)
	“I enjoyed the Zoom… I almost prefer the Zoom because it’s coming home to me and not having to worry where are we meeting, is it dark out, is there a safety issue when I go to leave the meeting because now it’s getting dark” (participant #3)
Receptiveness to study components	“The class is no longer available, but I still have the information [the workbook]” (participant #11)
	“[The text messages were a way] to keep focused and reminded” (participant #75)
	“I found that we didn’t have enough time to complete them [in class activities] in a thoughtful manner” (participant #94)
New additions	“I think that having a Facebook group or some sort of ongoing involvement with one another, breakout groups, or things during the session would have been helpful. Just to get to know the other participants a bit better” (participant #94)
	“more concrete recommendations…So you know to do so much resistance, for certain muscle types, muscle groups in the body” (participant #58)
	“even showing videos of, like inspirational videos of older people, like the progression you know? The first day they started, 30 days in. Just showing a snippet of that too, and then the types of exercises they’re doing” (participant #66)
	“usually like four of them would mainly do most of the talking” (facilitator 1—control group)

### Positive experiences

#### Group interaction/common humanity

Participants appreciated being in the programme with people who also experienced similar challenges with engaging in physical activity. Discussions within the group sessions highlighted for participants that they are not alone and that they are human and make mistakes. Some participants noted that a benefit of recognizing that everyone experiences challenges related to physical activity is that this motivated them to work harder after experiencing a physical activity setback. Some participants also discussed how everyone was very positive and supportive within the group, which helped them have a positive experience overall. These sentiments provided by participants highlight how having group interaction throughout the study was a positive experience.

#### Changed perspective and understandings

Participants’ outlooks on physical activity changed. For some participants, this was manifested by recognizing they were in control of their situation and could enact change in their lives. Similarly, participants’ outlook on physical activity as it relates to health changed and physical activity came to be seen as a tool they could use to take control of their health. For instance, participants realized that small bouts of exercise can be beneficial to their health (both their mind and their body). Others noted a changed perspective on how they viewed physical activity setbacks; they did not view setbacks through a failure lens but with hope. These changed perspectives also had positive effects on participants’ motivation to continue to engage in physical activity and were seen as a benefit to being in the programme.

#### Changed behaviour

Many participants noted a change in their health behaviour engagement. For many, this change manifested in increasing their physical activity levels. Others focused on other health behaviours, such as reducing sedentary time, as they viewed these changes as small, achievable steps they could do to improve their health and diabetes risk. After making changes to their behaviours, some participants noted and were excited that they had reaped some of the benefits of making healthy lifestyle choices. Lastly, changed behaviour also took the form of improvements in how some participants treated themselves; they were kinder and more mindful of what they needed in order to be healthy. Taken together, a positive experience that many participants took from this study was noticeable behaviour change that should contribute to their health and well-being.

### Receptiveness

#### Receptiveness to session content

Response to both the intervention and control session contents was generally positive. Participants enjoyed learning about behaviour change strategies such as SMART goal setting; they recognized the positive impact that setting goals could have on their physical activity engagement. Participants in the self-compassion condition recognized the value of relating to themselves with self-compassion yet also expressed difficulty with implementing this skill. For instance, some participants commented on their lack of familiarity with self-compassion but felt that they could see themselves implementing this approach in the future. Others found some of the self-compassion exercises awkward and difficult to implement. Overall, it appeared most participants saw the value of self-compassion despite it being challenging to implement at times. Participants in the control group felt they already knew some of the session material but stated they found it to be a good reminder. Others in the group found the information particularly helpful with regard to their diabetes management. Overall, the control material was also generally very well received.

#### Receptiveness to structure and format

Participants thought the structure and format of the online sessions were good. Generally, they were happy with the length and time of day of the sessions and emphasized that they went by quickly. Yet, many participants said they would have preferred more sessions. In addition, two out of the five sessions ran over the allotted time; some participants mentioned they would have preferred that all sessions ended on time. Despite this challenge, both facilitators thought it would be important to make class sessions longer in the future trial in order to facilitate rich discussions. Many participants also spoke about the convenience, comfort, and anonymity benefits of conducting the study online—although a few stated they would have preferred in-person to build more of a connection with the other group members.

#### Receptiveness to study components

Participants provided assurance that the components of the study (e.g. text messages, website) were valuable and tolerable. The website was well-received, though many participants expressed that they did not use the website as often as they intended. Workbook and home practices were mostly enjoyed; participants particularly liked having a book that they could refer to outside of class. Participants found both the online questionnaires and wearing the accelerometer tolerable. In addition, the text messages were considered a great way to remember the aspects of the study. Participants also suggested some components they would change. For instance, participants thought having more time to complete the workbook activities in class would be beneficial.

### New additions or changes

The facilitators and some participants offered new ideas that could supplement our planned future study. Participants expressed interest in making connections with the group outside of the weekly meetings such as by creating a Facebook group or doing an exercise class together. Other participants identified that it would be useful to include more specific physical activity recommendations (e.g. what type of exercise they should be doing). Some ideas for helping other participants increase their physical activity engagement, such as by showing inspirational videos of older people increasing their physical activity, were offered by participants. Despite an appreciation of the group setting, a challenge encountered by facilitators was engaging all participants during the session. Both the control and the intervention facilitators mentioned that usually, it was the same handful of individuals who would participate in class discussions; other members of the group minimally participated.

## Discussion

We examined the feasibility and acceptability of a self-compassion informed intervention designed to help people with prediabetes increase physical activity. The goal of the planned intervention that, in the future, will be tested for efficacy will be to determine whether self-compassion training can supplement behaviour change education and lead to greater physical activity among people with prediabetes than behaviour change education plus attention (clinical trials #NCT04863235). This planned future efficacy trial will be resource- and time-intensive, so our test of feasibility and acceptability is practical [42]. Our findings reveal that our planned efficacy trial should be feasible with some alterations made to the study components.

### Recruitment and process time

The time required to recruit enough participants for a wave and to move participants through eligibility exceeded our criteria of 2–3 weeks, which may have been unrealistic. In the future efficacy trial, we will allow 7 to 8 weeks for recruitment and processing. Increasing the number of people screened should further help with the recruitment; we will try to attract more people by increasing the number of individuals who see information about the study on social media. Indeed, we observed that increased exposure led to increased study interest. Given that many individuals were deemed ineligible because they were too active, we will adjust our advertisement messaging to emphasize that we are looking for individuals who are inactive. We will also seek out traditional media exposure such as radio and television as this coverage has proven to supplement social media recruitment. To reduce our process time, we will seek two methods of contact (i.e. phone number, email address) and remind participants of the importance of responding to study requests in a timely manner.

### Fidelity and capacity

Fidelity reports by facilitators and the principal investigator yielded scores that met our criteria for fidelity suggesting that our team can adhere to the protocol. One feasibility issue with intervention delivery was that sessions 2 and 3 out of six went over time (for both groups). For the future efficacy trial, we will increase the session length for all sessions from 1.5 to 2 h which should be acceptable to participants as many expressed a desire for longer sessions (discussed in more detail later). We also found that the number of hours relative to the budgeted cost for study personnel stayed within the appropriate parameters throughout the study duration.

### Attendance, adherence, and acceptability

Participants consistently attended study sessions (98.93%), and this attendance met our criterion. Importantly, we had high retention and adherence in both our intervention and control conditions, which we see as important; retention and adherence to control conditions are a challenge for interventionists [31]. We attribute our high retention, adherence, and acceptability of intervention content to a general sense by study personnel and participants in both conditions that the sessions were enjoyable and value-laden. Given the focus of the efficacy trial on testing the additive value of self-compassion when used to augment behaviour change training, all participants were presented with tangible information with the potential to be practically valuable to them. Indeed, all participants received behaviour change training and either other health information (control group) or self-compassion training (intervention group), and each of these types of content was reported as being valuable and interesting. We were further encouraged that participants were accepting of self-compassion content given that fear or rejection of self-compassion has been documented [60]. Our participants’ acceptance of self-compassion training aligns with past feasibility research where self-compassion training was also accepted by participants with chronic health conditions [48]. This can further inform other research studies when using self-compassion training among individuals diagnosed with a chronic disease.

The convenience of our online format expressed by participants likely contributed to our high adherence rates. This pilot study took place during a time when COVID-19 restrictions advised people to stay at home as much as possible and prohibited group gatherings, so the online option gave them a safe way to participate. Further, participants noted the convenience of this format for times when they are feeling tired or trying to get to a session during a busy day. The acceptability of the online format aligns with previous research studies that also report the proficiency and acceptability of online psychological interventions among individuals with diabetes [61] and chronic diseases [62]. This further aligns with online self-compassion interventions that have also been deemed acceptable with many advantages [63–65]. Researchers even request the need for in-person interventions to be adapted to an online format among teenagers with type 1 diabetes [66]. Overall, the present study offers further support for the acceptability of an online self-compassion intervention.

Participants also appreciated the aspects of the study put in place to support the intervention: the study website, workbooks, and reminder text messages. For instance, participants appreciated having the workbook after the sessions ended as it reminded them of what they needed to do to increase their physical activity. However, there was a sense from some participants that they underused the website. Many participants mentioned they forgot about the website, were too busy, or felt overwhelmed by the amount of information on the website. Although the lack of time is out of our control, it seems to be a common barrier to accessing online resources [63]. In our future efficacy trial, we will highlight related website content each week during the sessions to remind participants about this resource.

Despite many participants noting that home practice was acceptable and helped keep the information in the forefront of their minds, adherence to home practice did not meet our criterion and requires changes. In order to address this shortcoming, in our efficacy trial, we will remind participants at each session of the importance of completing the home practice. In addition, at the start of each session, we will ask the participants about their experience with the home practice in order to make home practice salient and to foster a sense of accountability.

Retention of participants to the study assessments (i.e. questionnaires; accelerometer-wear) met our criteria. Further, participants found questionnaires easy to understand and not burdensome. Examination of the means revealed an error in our wording of the IPAQ that we can fix for the main efficacy trial (i.e. we used the word bouts instead of days when asking about physical activity). Participants also found wearing accelerometers to be acceptable and easy to understand. Evidence that wearing an accelerometer should be feasible is important as physical activity will be the main outcome variable of the planned efficacy trial. Taken together, we anticipate that it most likely will be feasible to retain participants within the future efficacy trial and that we can expect high adherence to the intervention and measurement aspects.

### Suggested changes for efficacy trial

Despite the general feasibility and acceptability of the intervention, interviews with participants revealed more detail and suggested improvements. There was a desire by both participants and facilitators for more and/or longer sessions; as discussed previously, we will increase the number of intervention sessions from six to eight and the length of each session from 1.5 to 2 h. This change should alleviate the challenges with covering content and provide sufficient time for in-class activities and meaningful conversation by more participants. This should also prevent group sessions from going overtime.

In terms of actual content, we will modify, replace, or expand some topics based on feedback from participants in the interviews. In the future efficacy trial, within the behaviour change topic of enjoyment, we will acknowledge that for some people exercise may never be enjoyable, yet continue to suggest ways that it can be rendered more enjoyable. Indeed, some participants felt it was important to acknowledge that for some people, exercise may never be an enjoyable activity. We will replace the topic of antibiotic resistance (which was controversial) with a different health topic (e.g. blood pressure) and develop two additional control topics. We will not add a physical activity component to the intervention as requested by some participants as it would be difficult to find time and activity suitable for all participants. However, the longer duration of the sessions will allow more time during the first online one-on-one session for the research assistant to talk in-depth with the participant about the physical activity guidelines and the benefits of exercise and to explore the participants’ personal physical activity history and goals. Further, we will add a video with a testimonial from a past participant who has successfully become more active, as per the suggestion to add some video content.

Some participants suggested the addition of a forum for participants within a common intervention group to communicate, such as a Facebook page. We decided that due to ethical considerations of managing such a group, we will not formally provide this to participants. That being said, we will not prevent participants from creating their own Facebook group on their own initiative.

### Limitations and generalizability

We conducted our feasibility study when there were restrictive COVID-19 restrictions in place. For example, intervention sessions and follow-up assessments occurred during a time when all non-essential businesses were closed, and social gatherings were not allowed. These unusual circumstances may be different from the circumstances in place (fewer or no restrictions) when the efficacy trial is carried out. We speculate that participants in our pilot study may have had fewer competing demands on their time than if there had been less restrictive health order in place and this may have contributed to our high adherence and attendance rates. These rates may not represent those that we would attain had we completed the study in more typical times. We plan to begin our efficacy trial at a time when COVID-19 restrictions will likely be in place so there is likely value in having conducted our pilot work in a similar context. However, our findings may not generalize well to trials conducted when pandemic restrictions are not in place. In addition, the present study is not intended to generalize its findings to other research studies of similar nature [67]. However, there are aspects of our findings that may be beneficial for other researchers. Some examples of how our study can be generalizable include the feasibility of the online intervention delivery format; how it may be advantageous to create a control group with valuable material for participants to help with retention, accelerometer wear acceptability, and the acceptability of the intervention content (i.e. concept of self-compassion, behaviour change, and health behaviour information).

We took the recommended and informative [42] preliminary research step of examining the feasibility and acceptability of our planned intervention. Through this pilot feasibility and acceptability study, we learned that all aspects of our study should be feasible and acceptable. We also learned what steps we need to take to increase the likelihood of implementing a feasible and acceptable efficacy trial. In summary, we will give ourselves more time to recruit participants and complete all eligibility steps, stress the importance of home practice completion, provide an opportunity to discuss the home practice within class sessions, increase the duration and number of sessions, modify and add some intervention topics, add a testimonial from a past participant who has successfully become more active, and provide more personalized information on physical activity and goal setting. We are encouraged by the general feasibility and acceptability of our study and once these changes are implemented, we will proceed with testing the efficacy of a self-compassion intervention in augmenting traditional behaviour change training to bring about increases in physical activity among people with prediabetes.

## Supplementary Information

**Additional file 1: Table 1.** Resources shared with participants. **Table 2.** Interview Guide. **Table 3.** Means of Study Variables.

## Data Availability

The datasets used and/or analysed during the current study are available from the corresponding author on reasonable request.

## Footnotes

1 For this pilot study, we conducted the assessment at 5 weeks post-intervention rather than at 6 weeks as planned given that 6 weeks post-intervention week fell over Christmas (December 25) when people’s schedules were likely to be impacted by the holiday.
