# Supplementary material for: A pilot and feasibility study of a randomized clinical trial testing a self-compassion intervention aimed to increase physical activity behaviour among people with prediabetes
Source: Pilot Feasibility Stud. 2022 May 27;8:111. doi: 10.1186/s40814-022-01072-6 (PMC9135984; doi:10.1186/s40814-022-01072-6)
Supplement: Supplementary file 1 — Additional file 1: Table 1. Resources shared with participants. Table 2. Interview Guide. Table 3. Means of Study Variables. [file 40814_2022_1072_MOESM1_ESM.pdf]

**Table 1** Resources shared with participants

| PDF Articles | URL |
|---|---|
| Canadian Physical Activity Guidelines | https://csep.ca/CMFiles/Guidelines/CSEP_PAGuidelines_0-65plus_en.pdf |
| Diabetes Canada: Just the basics | https://guidelines.diabetes.ca/docs/patient-resources/just-the-basics-EN.pdf |
| Diabetes Canada: Managing weight and diabetes | https://guidelines.diabetes.ca/docs/patient-resources/managing-weight-and-diabetes.pdf |
| Tips to Get Active | https://www.canada.ca/en/public-health/services/publications/healthy-living/physical-activity-tips-adults-18-64-years.html |
| Diabetes Canada: Sugars and sweeteners | https://guidelines.diabetes.ca/docs/patient-resources/sugars-and-sweeteners.pdf |
| **Website** | **URL** |
| Intervention Group | https://www.umpachangestudy.com/ |
| Control Group | https://www.umprediabetesstudy.com/ |

**Table 2** Interview Guide

---

Hello, my name is ________ and I am the research assistant that will be interviewing you today!

Now, before we get started on the actual questions and formal interview process, I know we've communicated a lot over email the past few months, but It would be really nice to learn a bit more about you! Can you tell me a little bit about yourself?

- Age, occupation, family/social life
- What do you like to do in your spare time?

First, I just want to thank you once again for being willing to participate in the interview aspect of the MOVE IT study. As we have mentioned to you before, this study is to compare the effectiveness of two educational sessions both designed to help people living with prediabetes increase their physical activity. This was a test-run to see if we can implement this same study on a larger scale. Therefore, the aim of this exit-interview is to document your thoughts and feelings regarding your experience in the MOVE IT study.

Our interview today will last approximately one hour during which I will be asking you about your thoughts about different components of this study.

We sent you the consent form that you completed which indicated that I have your permission (or not) to audio record our conversation.
Are you still ok with me recording (or not) our conversation today? ___Yes ___No
If yes: Thank you! Please let me know if at any point you want me to turn off the recorder or keep something you said off the record. However, I will remind you that anything that you do say in this interview will not be linked to your name.

If no: Thank you for letting me know. I will only take notes of our conversation.

Although mentioned on the consent form, I would like to remind you that:

- Your name will not be used and instead we will be using a pseudo (pretend) name
- There are no right or wrong answers
- You do not have to answer any questions you do not want to answer
- If you have additional thoughts or questions after the interview, please do not hesitate to contact me via phone or email.
- You'll also have the opportunity to review your transcript for accuracy and modify or remove anything you choose, if you would like. Is this something you would like to do?
    - If yes: you can expect to receive your transcribed interview in the next 2-3 weeks.

Before we begin the interview, do you have any questions? [Discuss questions]

If any questions (or other questions) arise during the interview, you can feel free to ask them at any time. I would be more than happy to answer your questions.

Now I would like to talk to you a bit about the **online sessions** you attended. Please make sure you are honest in your answers - this information is extremely useful to us and can be used to guide future physical activity classes.

1. How did you first hear about the MOVE IT study?
2. What was your overall impression of the intervention?
3. What are your thoughts about the *number* of group sessions that were part of this study?
    a. Follow-up: Please explain whether you thought the number of online group sessions was manageable, wanted more? Less?
    b. Please explain your thoughts on the first one-on-one meeting?
       PROMT: Was it helpful or useful to you?
4. What are your thoughts about the *time of day* of the group sessions?
    a. PROMPT: please explain whether you had trouble attending the session on time, would a different time work better for you (if so, when)?
5. Please explain what your thoughts are about the *length of each session* (i.e. too long, too short, just right).
6. Please explain your experience with being involved in the group sessions through Zoom relative to meeting in person (if that was possible).
    a. PROMPT: Technical difficulties?, Benefits?, Drawbacks?
7. Please explain which topics, if any, you enjoyed learning about the most?
8. Please explain which topics, if any, you disliked learning about?
9. Please explain whether you felt like the topics covered helped you increase your physical activity levels.
    a. PROMPTS: What activities were the most helpful? Least helpful?
10. Please explain whether there was anything that could have helped you to further increase your physical activity that was not included in the group sessions?
11. Please explain any other benefits you gained from participating in the MOVE IT Study.
12. Please explain whether there was anything in the group sessions that made you feel comfortable, welcomed, or connected to the group.
    a. PROMTS: facilitator, topics, discussions, activities.
13. Please explain whether there was anything in the group sessions that made you feel uncomfortable, either physically or emotionally.
    a. PROMPTS: Topics, discussions, home practice, worksheet activities.

During each class, the facilitator had you complete **in-class activities in your workbook**. I am going to ask you a couple questions specifically related to these activities.

14. What was your overall impression of the in-class activities?
    a. PROMPT: Helpful/not helpful – why or why not.

    Please explain which workbook activity, if any, you enjoyed completing the most?
    Please explain which workbook activity, if any, you found not helpful/enjoyable.
15. Throughout the 5 weeks of classes, can you please explain whether if at any point you referred to your workbook on non-class days in order to help you increase your PA?
16. Throughout the last couple of weeks since the group sessions ended, please comment on whether or not you have referred back to your notes in the workbook to help you stay on track with your physical activity goals?

Wonderful! Thank you for answering those questions. Next, I am just going to ask you a few questions about the **home-practice**. Put some questions below.

1. What was your overall impression of the home practice activities that you were given?
   a) PROMPT: Helpful/not helpful – why or why not.
   b) PROMPT: Too much/too little

   Please explain which home practice, if any, you enjoyed completing the most.
   Please explain which home practice, if any, you found least enjoyable/helpful/relevant.
2. Can you explain whether it was easy or difficult to complete the home practice activities.
   a) PROMPT: How much home practice activities would you say you completed?
3. At the beginning of the study, we provided you with different resources that may be beneficial for you to review. For example, we provided you with a website that provided you with additional information about the topics covered in class. Please explain whether you used any of these resources.
   a) PROMPT: if you did/did not – please explain why.

Great. Thank you very much. We are going to switch gears a bit now and talk about the **online questionnaires** you completed before and after the intervention (as well as you will be completing at the 6- and 12-week follow-up time points).

1. What was your overall impression of the online questionnaires?
2. How long, on average, did it take you to complete the online questionnaires?
3. How do you feel about the length of time it took to complete the questionnaires?
4. Please explain whether you had any troubles accessing and/or filling out the questionnaires?

The next topic we are going to discuss is about the **text messaging system**. As we mentioned in the beginning of the study, this is our first time piloting this system and so we are open to any feedback (positive or negative) you have.

1. What are your general thoughts about the text messaging system used as part of the MOVE IT study?
2. What are your thoughts about the *number* of texts received?
3. Please explain whether you thought the texts messages throughout the study were helpful/not helpful to you.
   a. If applicable, please explain **how** these texts were helpful to you during the study.
   b. If applicable, please explain **why** these texts were not helpful to you during the study.

Now I would like to ask you a few questions about the **accelerometer** (the red device that sat on your right hip and tracked your movement throughout the day) that you wore both before the study and after the group sessions (*remember not all participants wore them – so only ask this for those who did).

1. Please explain how you felt about having to wear it for the 8 days.
2. Did you have any difficulty remembering to put it on?

   a. If so, please explain
3. Please explain whether the accelerometer tracking sheet was understandable to you and whether or not you had any troubles filling it out?
4. Please explain whether you had any difficulties remembering to put the accelerometer on in the morning
5. Are there any other thoughts, feelings, or general comments about the study that you would like to share before we complete today's interview?

Great – Thank you so much for allowing me to ask you all of these questions today and thank you for being so open in sharing your experience with me. Receiving your feedback is a vital component of our study so that we can work on improving it for the future trial.

**Table 3** Means of Study Variables

| | Control | | | | | | | | |
|---|---|---|---|---|---|---|---|---|---|
| | Scale Range | Baseline (n = 8) | | Post-Intervention (n = 8) | | 6 -Week Follow-Up (n = 7) | | 12 -Week Follow-Up (n = 7) | |
| **Variable** | | **Mean** | **SD** | **Mean** | **SD** | **Mean** | **SD** | **Mean** | **SD** |
| Self-Compassion | 1 to 5 | 2.90 | 0.61 | 3.26 | 0.73 | 3.23 | 0.68 | 3.25 | 0.75 |
| Exercise Barriers | 14 to 46 | 31.00 | 9.05 | 29.75 | 9.07 | n/a | n/a | n/a | n/a |
| Diabetes Affect | | | | | | | | | |
| Sadness | 4 to 28 | 17.25 | 8.33 | 15.00 | 8.96 | 15.86 | 7.47 | 14.14 | 7.84 |
| Anxiety | 4 to 28 | 18.25 | 7.19 | 16.75 | 9.03 | 16.00 | 8.43 | 16.28 | 8.20 |
| Anger | 4 to 28 | 12.25 | 7.13 | 12.50 | 7.35 | 11.14 | 6.67 | 10.57 | 5.86 |
| Embarrassment | 4 to 28 | 15.87 | 9.06 | 13.00 | 8.42 | 13.14 | 7.65 | 11.14 | 6.69 |
| Incompetence | 4 to 28 | 16.87 | 7.62 | 14.25 | 8.15 | 13.00 | 6.86 | 11.57 | 4.50 |
| Physical Activity Affect | | | | | | | | | |
| Sadness | 4 to 28 | 14.87 | 6.47 | 15.25 | 8.19 | 13.43 | 6.11 | 11.14 | 6.44 |
| Anxiety | 4 to 28 | 14.75 | 7.65 | 14.12 | 9.00 | 12.28 | 7.27 | 11.57 | 7.00 |
| Anger | 4 to 28 | 10.25 | 7.68 | 10.87 | 6.68 | 6.57 | 3.78 | 7.28 | 3.82 |
| Embarrassment | 4 to 28 | 14.50 | 10.17 | 15.00 | 8.14 | 12.86 | 7.20 | 8.43 | 3.73 |
| Incompetence | 4 to 28 | 15.00 | 8.18 | 16.25 | 9.18 | 12.57 | 6.92 | 11.57 | 6.32 |
| Health Promoting Behaviours | | | | | | | | | |

| | | | | | | | | | |
|---|---|---|---|---|---|---|---|---|---|
| Health Responsibility | 1 to 4 | 2.68 | 0.72 | 2.87 | 0.72 | 2.85 | 0.73 | 2.92 | 0.81 |
| Physical activity | 1 to 4 | 1.84 | 0.96 | 2.01 | 0.67 | 2.30 | 0.93 | 2.46 | 0.77 |
| Nutrition | 1 to 4 | 2.60 | 0.61 | 2.80 | 0.55 | 2.81 | 0.63 | 2.87 | 0.58 |
| Spiritual Growth | 1 to 4 | 2.55 | 0.64 | 2.61 | 0.58 | 2.52 | 0.66 | 2.71 | 0.61 |
| Interpersonal Relations | 1 to 4 | 2.93 | 0.54 | 2.90 | 0.63 | 2.80 | 0.74 | 2.98 | 0.61 |
| Stress Management | 1 to 4 | 2.42 | 0.66 | 2.50 | 0.53 | 2.34 | 0.64 | 2.55 | 0.51 |
| CERQ | | | | | | | | | |
| Self-blame | 4 to 20 | 11.25 | 2.76 | 11.75 | 4.74 | 11.00 | 2.94 | 9.43 | 0.97 |
| Acceptance | 4 to 20 | 10.62 | 2.13 | 10.12 | 3.18 | 9.86 | 3.44 | 10.14 | 1.95 |
| Ruminate | 4 to 20 | 12.75 | 3.88 | 11.37 | 4.24 | 10.57 | 3.69 | 11.57 | 3.60 |
| Positive Refocusing | 4 to 20 | 8.75 | 2.43 | 10.87 | 4.67 | 9.14 | 2.27 | 11.71 | 3.99 |
| Refocus on planning | 4 to 20 | 11.87 | 3.64 | 13.50 | 4.50 | 11.14 | 3.48 | 12.71 | 4.19 |
| Positive Reappraisal | 4 to 20 | 13.12 | 4.39 | 14.00 | 4.87 | 12.57 | 4.82 | 14.43 | 4.58 |
| Perspective | 4 to 20 | 14.62 | 4.81 | 13.25 | 5.15 | 13.28 | 5.15 | 12.57 | 3.05 |
| Catastrophizing | 4 to 20 | 6.87 | 2.69 | 7.00 | 3.66 | 7.57 | 4.08 | 7.71 | 3.25 |
| Blaming Others | 4 to 20 | 6.75 | 2.05 | 6.00 | 1.60 | 6.14 | 2.19 | 5.57 | 1.81 |
| METS Walk | | 168.30d | 157.85 | 439.31d | 402.33 | 170.50b | 176.07 | 499.71c | 476.10 |

| | | | | | | | | | |
|---|---|---|---|---|---|---|---|---|---|
| METS Moderate | | 127.50d | 188.21 | 182.5d | 255.10 | 220.00c | 172.43 | 328.57c | 319.55 |
| METS Vigorous | | 0.00d | 0.00 | 130.00d | 190.94 | 629.71c | 929.11 | 160.00c | 236.64 |
| Total METS week | | 295.80d | 278.35 | 751.81d | 476.42 | 721.83b | 801.86 | 988.29c | 389.12 |
| Light PA per week (GT3X+)** | | 1673.00 | (1377.00, 2323.42) | 1543.00 | (1345.62, 2051.50) | 1791.00 | (1333.37, 2287.25) | 1692.00 | (1394, 1999.75) |
| Moderate PA per week (GT3X+)** | | 28.00 | (19.00, 176.00) | 59.50 | (33.00, 262.50) | 90.75 | (19.12, 210.00) | 126.00 | (15.00, 399.00) |
| Vigorous PA per week (GT3X+)** | | 0.00 | 0.00 | 0.00 | (0.00, 0.87) | 0.00 | 0.00 | 0.00 | 0.00 |
| Steps per day (GT3X+) | | 3671.51 | 1222.45 | 3728.75 | 1149.01 | 5305.81 | 2846.38 | 6315.64 | 3982.82 |
| Self-Kindness | 1 to 5 | 3.12 | 0.80 | 3.17 | 0.80 | 3.14 | 0.79 | 3.03 | 0.65 |
| Common Humanity | 1 to 5 | 3.22 | 0.56 | 3.34 | 0.56 | 3.39 | 1.07 | 3.21 | 0.68 |
| Mindfulness | 1 to 5 | 3.22 | 0.59 | 3.59 | 0.59 | 3.28 | 0.60 | 3.28 | 0.65 |
| Self-Judgement | 1 to 5 | 3.45 | 0.63 | 2.94 | 1.16 | 2.97 | 0.71 | 2.86 | 0.76 |
| Isolation | 1 to 5 | 3.12 | 1.21 | 2.69 | 1.09 | 2.53 | 1.00 | 2.61 | 1.30 |
| Over-Identification | 1 to 5 | 3.41 | 1.23 | 2.90 | 0.68 | 2.93 | 0.96 | 2.57 | 0.95 |

**Intervention**

| | **Scale Range** | **Baseline (n = 7)** | | **Post-Intervention (n = 7)** | | **6 -Week Follow-Up (n = 7)** | | **12 -Week Follow-Up (n = 7)** | |
|---|---|---|---|---|---|---|---|---|---|
| **Variable** | | **Mean** | **SD** | **Mean** | **SD** | **Mean** | **SD** | **Mean** | **SD** |
| Self-Compassion | 1 to 5 | 2.94 | 0.29 | 3.12 | 0.72 | 3.18 | 0.55 | 3.19 | 0.56 |

| | | | | | | | | | |
|---|---|---|---|---|---|---|---|---|---|
| Exercise Barriers | 14 to 46 | 34.58 | 2.22 | 30.15 | 3.93 | n/a | n/a | n/a | n/a |
| Diabetes Affect | | | | | | | | | |
| Sadness | 4 to 28 | 14.28 | 5.91 | 11.86 | 5.05 | 12.57 | 5.41 | 12.00 | 4.43 |
| Anxiety | 4 to 28 | 14.71 | 4.99 | 13.00 | 5.74 | 13.71 | 5.28 | 13.43 | 4.86 |
| Anger | 4 to 28 | 7.86 | 3.44 | 10.95 | 8.60 | 9.14 | 7.20 | 11.00 | 6.00 |
| Embarrassment | 4 to 28 | 12.28 | 5.09 | 10.28 | 5.19 | 13.14 | 6.39 | 10.71 | 4.39 |
| Incompetence | 4 to 28 | 12.00 | 4.58 | 9.71 | 5.99 | 11.14 | 4.74 | 9.14 | 4.02 |
| PA Affect | | | | | | | | | |
| Sadness | 4 to 28 | 13.86 | 6.96 | 11.43 | 5.71 | 13.57 | 5.79 | 12.57 | 6.02 |
| Anxiety | 4 to 28 | 15.28 | 4.46 | 12.57 | 5.62 | 12.43 | 5.56 | 11.14 | 4.37 |
| Anger | 4 to 28 | 8.14 | 4.81 | 10.00 | 5.86 | 10.14 | 4.49 | 9.86 | 3.39 |
| Embarrassment | 4 to 28 | 15.00 | 5.69 | 10.71 | 4.96 | 12.28 | 5.88 | 13.86 | 6.96 |
| Incompetence | 4 to 28 | 13.86 | 5.43 | 11.86 | 5.96 | 12.14 | 3.85 | 11.57 | 5.47 |
| Health Promoting Behaviours | | | | | | | | | |
| Health Responsibility | 1 to 4 | 2.17 | 0.28 | 2.31 | 0.41 | 2.33 | 0.67 | 2.28 | 0.67 |
| Physical activity | 1 to 4 | 1.39 | 0.38 | 1.89 | 0.45 | 1.93 | 0.54 | 2.02 | 0.87 |
| Nutrition | 1 to 4 | 2.38 | 0.56 | 2.52 | 0.67 | 2.67 | 0.62 | 2.62 | 0.66 |
| Spritual Growth | 1 to 4 | 2.40 | 0.47 | 2.63 | 0.49 | 2.65 | 0.58 | 2.65 | 0.67 |
| Interpersonal Relations | 1 to 4 | 2.62 | 0.58 | 2.71 | 0.52 | 2.63 | 0.54 | 2.71 | 0.62 |

| | | | | | | | | | |
|---|---|---|---|---|---|---|---|---|---|
| Stress Management | 1 to 4 | 2.16 | 0.32 | 2.52 | 0.23 | 2.63 | 0.54 | 2.55 | 0.48 |
| CERQ | | | | | | | | | |
| Self-blame | 4 to 20 | 11.28 | 3.54 | 9.86 | 2.11 | 9.43 | 1.27 | 11.43 | 3.69 |
| Acceptance | 4 to 20 | 9.28 | 1.11 | 9.57 | 1.51 | 10.14 | 2.34 | 9.86 | 2.73 |
| Ruminate | 4 to 20 | 11.28 | 2.14 | 9.86 | 3.29 | 12.86 | 2.97 | 8.29 | 2.43 |
| Positive Refocusing | 4 to 20 | 7.43 | 1.72 | 8.71 | 2.43 | 9.86 | 3.39 | 9.28 | 2.06 |
| Refocus on planning | 4 to 20 | 9.28 | 1.80 | 10.42 | 4.54 | 11.57 | 1.81 | 10.14 | 2.79 |
| Positive Reappraisal | 4 to 20 | 11.00 | 4.55 | 12.71 | 4.82 | 12.71 | 4.31 | 12.00 | 5.03 |
| Perspective | 4 to 20 | 11.86 | 4.26 | 13.29 | 4.71 | 13.71 | 3.90 | 11.43 | 3.95 |
| Catastrophizing | 4 to 20 | 6.28 | 1.38 | 6.57 | 1.81 | 6.71 | 1.50 | 6.28 | 2.50 |
| Blaming Others | 4 to 20 | 8.28 | 1.60 | 7.28 | 2.81 | 7.71 | 1.89 | 6.86 | 2.85 |
| METS Walk | | 207.43c | 205.83 | 491.04a | 545.02 | 262.02a | 309.77 | 227.15b | 198.11 |
| METS Moderate | | 137.14c | 130.35 | 751.43c | 1288.30 | 324.57c | 379.02 | 86.67b | 114.31 |
| METS Vigorous | | 51.43c | 94.42 | 217.14 c | 283.65 | 457.14c | 1209.49 | 866.67b | 2045.51 |
| Total METS week | | 396.00c | 212.57 | 1607.04a | 1575.02 | 1308.42a | 1488.23 | 1180.48b | 2164.14 |
| Light PA per week (GT3X+)** | | 1665.00 | (1370.54, 2366.12) | 1539.20 | (1084.00, 1836.25) | 1865.00 | (1049.00, 2312.33) | 1279.00 | (1177.00, 2356.50) |
| Moderate PA per week (GT3X+)** | | 35.12 | (14.25, 55.46) | 11.78 | (5.75, 85.00) | 29.00 | (6.00, 181.00) | 28.50 | (14.62, 116.50) |

| | | | | | | | | | |
|---|---|---|---|---|---|---|---|---|---|
| Vigorous PA per week (GT3X+)** | | 0.00 | (0.00, 0.00) | 0.00 | (0.00, 0.00) | 0.00 | (0.00, 1.00) | 0.00 | (0.00, 0.00) |
| Steps per day (GT3X+) | | 3911.23 | 1801.73 | 3478.92 | 2325.78 | 4540.46 | 3311.93 | 3681.73 | 2720.35 |
| Self-Kindness | 1 to 5 | 2.77 | 0.67 | 2.77 | 0.79 | 3.08 | 0.61 | 2.91 | 0.94 |
| Common Humanity | 1 to 5 | 3.03 | 0.87 | 3.07 | 1.16 | 3.28 | 0.96 | 3.21 | 1.07 |
| Mindfulness | 1 to 5 | 3.03 | 0.57 | 3.36 | 0.70 | 3.46 | 0.67 | 3.43 | 0.69 |
| Self-Judgement | 1 to 5 | 2.86 | 0.47 | 2.58 | 0.89 | 2.91 | 0.50 | 2.74 | 0.54 |
| Isolation | 1 to 5 | 3.32 | 0.55 | 3.07 | 0.97 | 3.14 | 0.75 | 2.82 | 0.66 |
| Over-Identification | 1 to 5 | 3.00 | 0.38 | 2.83 | 0.51 | 2.68 | 0.53 | 2.82 | 0.55 |

**Note*. Sample sizes for each analysis are denoted next to the means. a. 5 participants; b. 6 participants; c. 7 participants; d. 8 participants. ** Medians, and lower and upper quartiles are presented.
